# Children with perinatally acquired HIV exhibit distinct immune responses to 4CMenB vaccine

**DOI:** 10.1172/jci.insight.177182

**Published:** 2024-05-22

**Authors:** Nicola Cotugno, Alessia Neri, Marco Sanna, Veronica Santilli, Emma Concetta Manno, Giuseppe Rubens Pascucci, Elena Morrocchi, Donato Amodio, Alessandra Ruggiero, Marta Luisa Ciofi degl Atti, Irene Barneschi, Silvia Grappi, Ilaria Cocchi, Vania Giacomet, Daria Trabattoni, Giulio Olivieri, Stefania Bernardi, Daniel O’Connor, Emanuele Montomoli, Andrew J. Pollard, Paolo Palma

**Affiliations:** 1 Clinical and Research Unit of Clinical Immunology and Vaccinology, Bambino Gesù Children’s Hospital, IRCCS, Rome, Italy.; 2 Department of Systems Medicine and; 3 PhD Program in Immunology, Molecular Medicine and Applied Biotechnology, University of Rome “Tor Vergata,” Rome, Italy.; 4 Department of Neuroscience, Biomedicine and Movement Sciences, University of Verona, Verona, Italy.; 5 Unit of Epidemiology, Clinical Pathways and Clinical Risk, Medical Direction, Bambino Gesù Children’s Hospital, IRCCS, Rome, Italy.; 6 VisMederi Life Sciences Srl, Siena, Italy.; 7 Paediatric Infectious Disease Unit, “Luigi Sacco” Hospital, and; 8 Department of Biomedical and Clinical Sciences, University of Milan, Milan, Italy.; 9 Oxford Vaccine Group, Department of Paediatrics, University of Oxford and the NIHR Oxford Biomedical Research Centre, Oxford, UK.; 10 Department of Molecular and Developmental Medicine, University of Siena, Siena, Italy.

**Keywords:** AIDS/HIV, Vaccines, Adaptive immunity, Bacterial vaccines

## Abstract

Children with perinatally acquired HIV (PHIV) have special vaccination needs, as they make suboptimal immune responses. Here, we evaluated safety and immunogenicity of 2 doses of 4-component group B meningococcal vaccine in antiretroviral therapy–treated children with PHIV and healthy controls (HCs). Assessments included the standard human serum bactericidal antibody (hSBA) assay and measurement of IgG titers against capsular group B *Neisseria meningitidis* antigens (fHbp, NHBA, NadA). The B cell compartment and vaccine-induced antigen-specific (fHbp^+^) B cells were investigated by flow cytometry, and gene expression was investigated by multiplexed real-time PCR. A good safety and immunogenicity profile was shown in both groups; however, PHIV demonstrated a reduced immunogenicity compared with HCs. Additionally, PHIV showed a reduced frequency of fHbp^+^ and an altered B cell subset distribution, with higher fHbp^+^ frequency in activated memory and tissue-like memory B cells. Gene expression analyses on these cells revealed distinct mechanisms between PHIV and HC seroconverters. Overall, these data suggest that PHIV presents a diverse immune signature following vaccination. The impact of such perturbation on long-term maintenance of vaccine-induced immunity should be further evaluated in vulnerable populations, such as people with PHIV.

## Abstract 2

4CMenB vaccination in patients with ART-treated perinatally acquired HIV and healthy control individuals shows good safety, B cell subset alteration, and distinct immune signatures.

## Graphical abstract

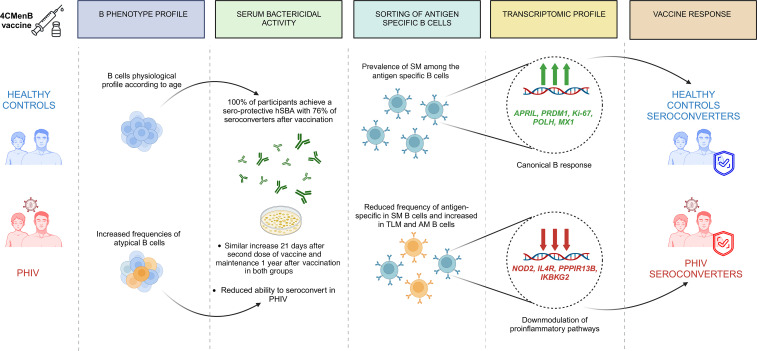

## Introduction

Globally, *Neisseria meningitidis* (*N*. *meningitidis*) is responsible for an estimated 1.2 million cases of invasive meningococcal disease (IMD) and 135,000 deaths each year (1). Serogroup B meningococcal (MenB) disease occurs globally and is endemic in Europe and Latin America (2). An increased risk of invasive infection with *N*. *meningitidis* is reported for individuals with certain types of immune deficiency or suppression, particularly for those with congenital complement deficiencies or those undergoing complement-neutralizing antibody therapy with eculizumab, but also those with asplenia or hypoglobulinemia (3–5). Adults living with HIV who meet AIDS surveillance criteria have a significant increase in the risk of meningococcal disease, compared with individuals who do not meet the criteria of AIDS (6). In this population, a US study found a 10-fold increased risk of IMD (7), and a study from South Africa reported that people living with HIV (PLWH) had an 11.3-fold increased risk of IMD (8). Hence, the development of a 4-component group B meningococcal (4CMenB) vaccine (Bexsero, GlaxoSmithKline [GSK]) and the MenB:factor H-binding protein (fHbp) vaccine (Trumenba, Pfizer) represents a significant advance in prevention of IMD, specifically for children with perinatally acquired HIV (PHIV) (9). The 4CMenB vaccine comprises 3 recombinant protein antigens, namely neisserial adhesin A (NadA), neisserial heparin binding antigen (NHBA), and fHbp, along with outer membrane vesicles expressing porin A protein (PorA). These components collectively offer broad protection against the majority of IMD caused by MenB strains (10, 11). In addition, anti–group B *N*. *meningitidis* vaccines have been recently shown to provide a cross-protective immune response against *N*. *gonorrhoeae* (12, 13). Therefore, administering 4CMenB vaccination to individuals with PHIV is a priority, not only from an individual but also from a public health perspective, as it contributes to the reduction of gonorrhea cases in this population. Few studies have assessed the immunogenicity and safety of meningococcal vaccines in PLWH (14), with defined molecular correlates of immune response still missing. Herein, we present a phase IV open-label vaccine study (MENIAC Study) aiming to investigate the safety, immunogenicity, and long-term memory maintenance (1 year) of 2 doses of 4CMenB vaccine in PHIV compared with healthy control (HC). B cell compartment and antigen-specific (Ag-specific) B cell transcriptional characteristics were investigated to define molecular signatures of suboptimal vaccine-induced immunity in this population.

## Results

A total of 63 people with PHIV aged 2–29 years (arithmetic mean ± SD: μ = 15,58 ± 5.84) and 28 HCs aged 3–26 years (μ = 10,96 ± 4.83) were enrolled in the study between September 2017 and November 2020. No differences were found in terms of sex between the groups, whereas patients with PHIV were significantly older compared with HCs (*P* < 0.001). Among the patients with PHIV, 62 individuals (98%) had an undetectable viral load (HIV copies [cps]/mL < 40), all individuals had < 200 cps/mL at baseline, and the mean CD4^+^ T cell percentage was 37.38% (±6.64%) (Table 1).

### Safety.

All solicited reactions were mild to moderate in severity and resolved within 3 days of onset. No severe local or systemic reactions were reported in both groups. Almost all participants (92.3% in HC group and 100% in PHIV group) reported at least 1 local and/or systemic reaction within 7 days after either vaccination. Most of these reactions were mild (grade 1), except 1 moderate (grade 2) in the PHIV group. The most frequently reported local reaction after any vaccination was local pain (92% for HC group and 95% for PHIV group), followed by erythema (53% for HC group and 55% for PHIV group), induration (46% for HC group and 45% for PHIV group), and swelling (38% for HC group and 40% for PHIV group). The most frequently reported solicited systemic reactions were malaise, headache, and muscle pain (31% for HC group and 30% for PHIV group), followed by nausea (16% for HC group and 15% for PHIV group). Fever ≥ 38°C was occasionally reported (23% for HC and 24% for PHIV group). No death, serious adverse event, or pregnancy occurred over the study.

### Two doses of 4CMenB vaccine elicit serological responses in PHIV and HC participants.

**Figure 1:**
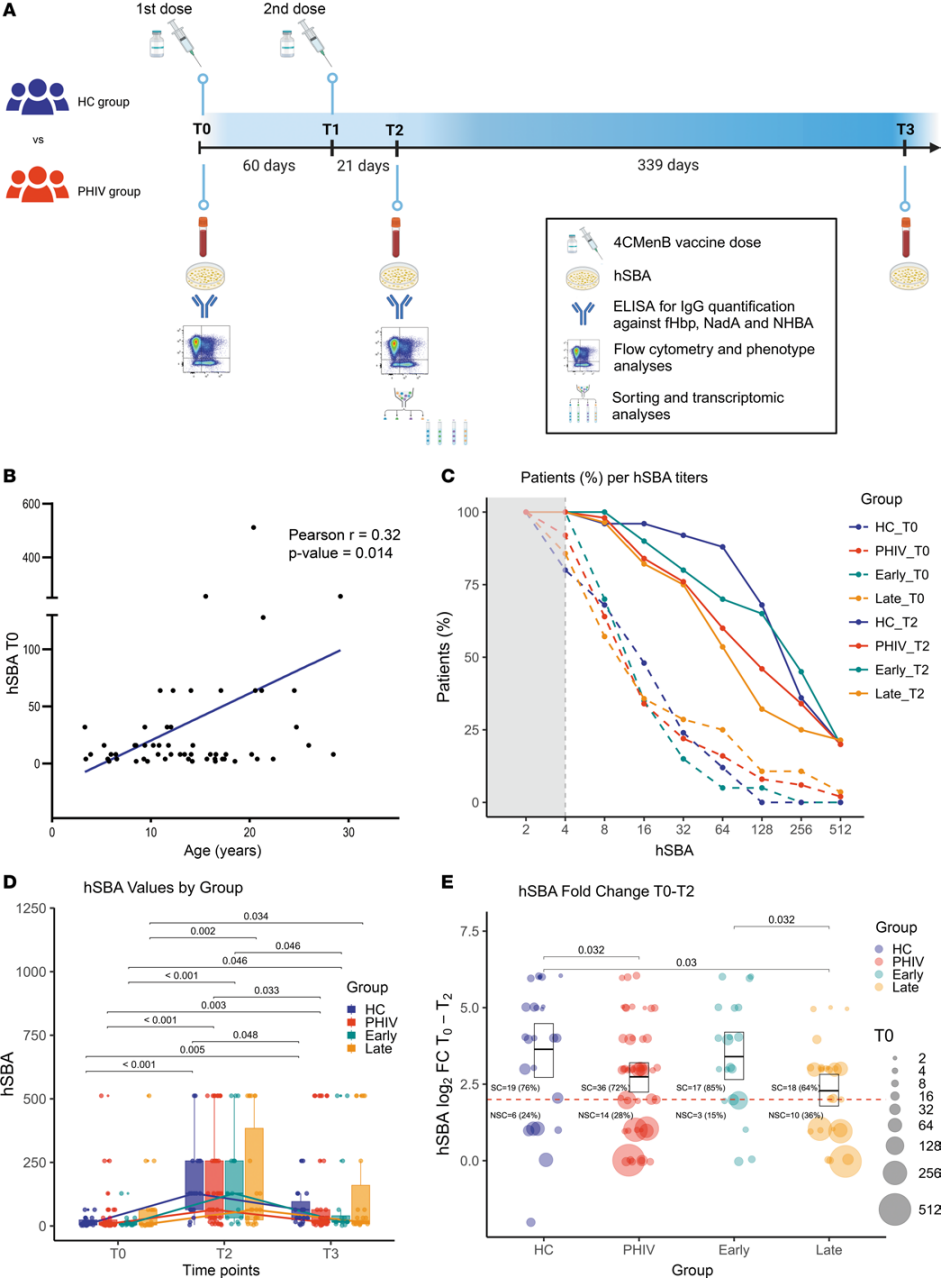
4CMenB vaccine elicits hSBA response in PHIV and HC participants. hSBA titers were measured in the sera of participants at T0, T2, and T3. (**A**) Study design. (**B**) Pearsonʼs correlation analyses between baseline hSBA values and age at T0. Regression line is reported in blue (*n* = 63). (**C**) Line plot reporting the percentage of participants per hSBA level at T0 (dashed lines) and T2 (solid lines) for HC (*n* = 25, blue) and PHIV (*n* = 50, red) groups and early-treated (*n* = 20, cyan) and late-treated (*n* = 28, orange) PHIV individuals. Gray dashed line marks the threshold of seroprotection (hSBA T0 = 4), with gray area showing nonseroprotection. (**D**) Longitudinal analyses of hSBA titer (±95% CI) in HC (*n* = 19, blue), PHIV (*n* = 39, red), and early-treated (*n* = 16, cyan) and late-treated (*n* = 23, orange) individuals pre- (T0) and postvaccination (T2–T3). The lines represent the median values of HC (blue), PHIV (red), and early-treated (cyan) and late-treated (orange) individuals. (**E**) Log_2_ hSBA titer fold-change (FC) (±95% CI) in HC (*n* = 25, blue), PHIV (*n* = 50, red), and early-treated (*n* = 20, cyan) and late-treated (*n* = 28, orange) participants following vaccination. The size of the points represents the hSBA value at T0. The dashed line represents the threshold of protection (FC ≥ 4); numbers and percentage of seroconverter (SC) and nonseroconverter (NSC) participants are reported for each group. Box plot midlines report the median, with the upper and lower limits of the box being 75th and 25th percentile, respectively. Whiskers represent 1.5 IQR. Comparison of hSBA distributions between groups and time points was evaluated by 2-way ANOVA followed by pairwise *t* test if both distributions were normal, or conversely, with Kruskal-Wallis test followed by Dunn’s test. FDR-adjusted *P* values less than 0.05 are reported.

To characterize the vaccine-induced response, the human serum bactericidal antibody (hSBA) level, which is a well-established correlate of protection (15, 16), at 3 time points (T0, T2, and T3) was quantified (Figure 1A). The effect of age on the baseline hSBA level was verified by a positive association between age and hSBA in the entire cohort (*r* = 0.32, *P* = 0.014) (Figure 1B). These observations are in line with the higher likelihood of meningococcal exposure associated with age, as described in previous works (16, 17). To exclude the effect of age at enrollment, all results presented in the study were verified by age-matched analyses reported in the supplemental material (Supplemental Figure 1; supplemental material available online with this article; https://doi.org/10.1172/jci.insight.177182DS1). Following vaccination, both groups showed a significant increase in hSBA levels, indicating a robust humoral immune response to the vaccine (Figure 1C). At T0, 92% (46/50) of PHIV and 80% (20/25) of HC participants had a protective hSBA titer of ≥ 4 (χ^2^ *P* = 0.26) (Figure 1C).

At T2, all participants demonstrated a positive responsiveness to the vaccine, with 100% achieving a seroprotective hSBA titer of ≥ 4 after the second dose (18, 19) (Figure 1C). No statistically significant difference was observed in the hSBA levels between the PHIV and HC groups (Figure 1D). Following the administration of 2 doses of 4CMenB, both groups exhibited a significant increase in hSBA levels at T2, which was followed by a significant decay at T3 (Figure 1D). Comparable levels of hSBA were found between T2 and T3 in the PHIV group and the HC group (Figure 1D). However, the ability to seroconvert after vaccination, expressed as hSBA FC between T0 and T2 ≥ 4, was significantly lower in PHIV compared with HC (*P* = 0.032, Figure 1E). Such differences did not seem to have an impact on the proportions of SCs (hSBA FC ≥ 4), which were (19/25) 76% and (36/50) 72% in HC and PHIV, respectively (χ^2^ *P* = 0.93) (Figure 1E). The analyses were verified by ELISA on the available samples, 20 from PHIV and 13 from HC at T0 and T2, though no significant differences at T2 emerged between the groups. At baseline, IgG levels for NHBA were significantly higher in the PHIV group compared with HC (*P* = 0.026) (Supplemental Figure 2). To gain further insights on the impact of timing of antiretroviral therapy (ART) treatment initiation on the vaccine response, hSBA was evaluated in early- (<6 months) and late-treated patients (Figure 1, C–E). As in previous reports (20, 21), the majority of the NSCs (hSBA FC < 4) were found in the late-treated group (10 out of 13, 77%), suggesting a more favorable response upon vaccination in early-treated individuals (Figure 1E). In line with this, the late-treated participants exhibited a reduced hSBA FC compared with the counterpart (*P* = 0.032) and presented a lower seroconversion rate of (18/28) 64% versus (17/20) 85% found in early-treated PHIV (χ^2^ *P* = 0.21) (Figure 1E). Only the late-treated group presented a reduced hSBA FC when compared with HC (*P* = 0.03, Figure 1E).

### PHIV impact on Ag-specific B cell profile and B cell subset distribution.

**Figure 2:**
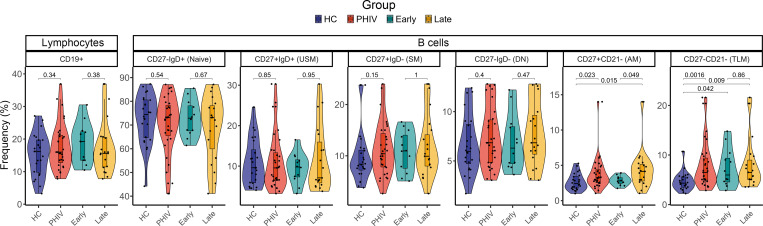
B cell subset frequency in PHIV and HC participants. The frequencies of B cell compartments (CD19^+^) were measured in PBMCs from PHIV and HC participants using flow cytometry at T2. Violin and box plots of the frequencies of total B cells (CD19^+^) and of the distinct B cell subsets (naive, unswitched memory [USM], switched memory [SM], double-negative [DN], activated memory [AM], tissue-like memory [TLM]) measured in PHIV (*n* = 31, red), HC (*n* = 24, blue), and early-treated (*n* = 12, cyan) and late-treated (*n* = 19, orange) PHIV participants. Box plot midlines report the median, with the upper and lower limits of the box being 75th and 25th percentile, respectively. Whiskers represent 1.5 IQR. Comparison of distributions between HC and PHIV was assessed by 2-tailed Mann-Whitney *U* test. Comparisons between HC and early- and late-treated individuals were assessed by pairwise *t* test when approximately normal or Dunn’s test conversely.

**Figure 3:**
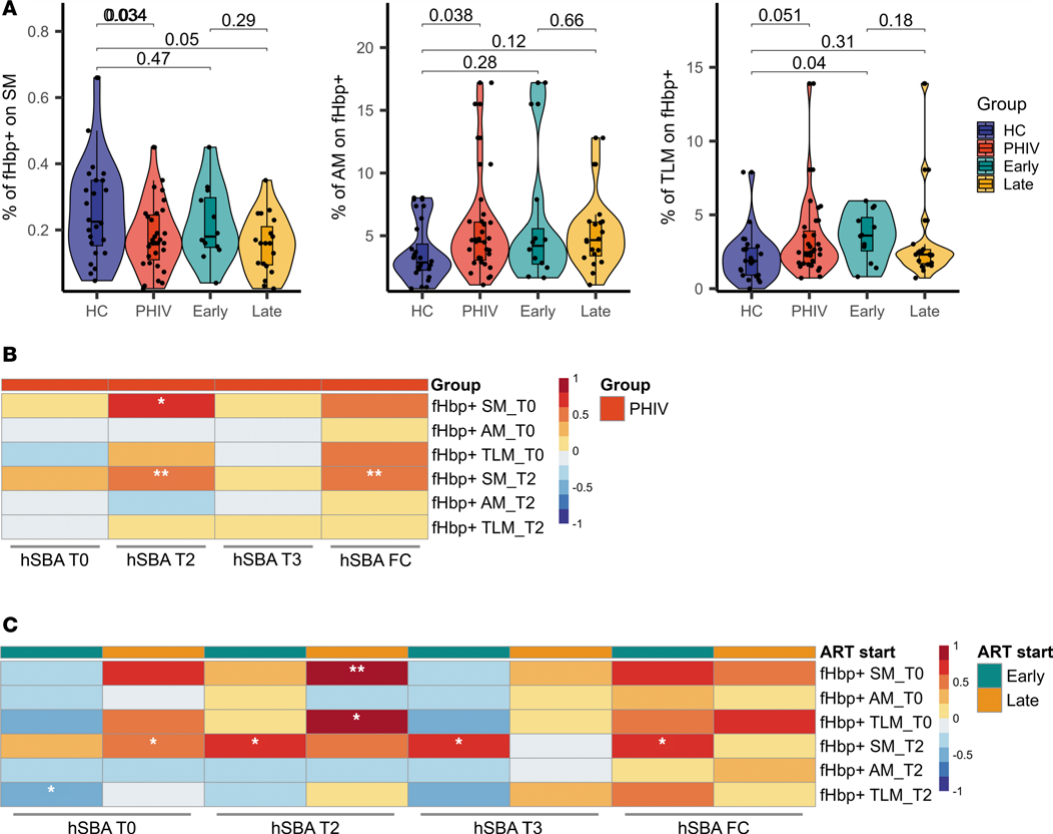
Ag-specific B cell subset distribution and immunological correlates in PHIV and HC participants. (**A**) Violin and box plots of the frequencies of Ag-specific (fHbp^+^) B cells measured in PHIV (*n* = 31, red), HC (*n* = 24, blue), and early-treated (*n* = 12, cyan) and late-treated (*n* = 19, orange) PHIV participants at T2. Box plot midlines report the median, with the upper and lower limits of the box being 75th and 25th percentile, respectively. Whiskers represent 1.5 IQR. Comparison between HC and PHIV was assessed by 2-tailed *t* test when approximately normal or 2-tailed Mann-Whitney *U* test conversely. Comparisons between HC and early- and late-treated individuals were assessed by pairwise *t* test when approximately normal or Dunn’s test conversely. (**B**) Pearsonʼs correlation heatmap between hSBA values at T0, T2, and T3, and fHbp^+^ cells at T0 and T2, for HC (*n* = 26) and PHIV (*n* = 59) groups. (**C**) Pearsonʼs correlation heatmap between hSBA values at T0, T2, and T3, and fHbp^+^ cells at T0 and T2, for early-treated (*n* = 21) and late-treated (*n* = 31) PHIV participants. *P ≤ 0.05, **P ≤ 0.01.

The B cell compartment and vaccine-induced B cell memory responses were evaluated by flow cytometry in the 2 groups at T2 (gating strategy reported in Supplemental Figure 3A). No differences emerged in terms of CD19^+^ B cells between PHIV and HC. However, a distinct B cell distribution with a significantly higher frequency of activated memory (AM: CD27^+^CD21^–^, *P* = 0.023) and tissue-like memory (TLM: CD27^–^CD21^–^, *P* = 0.0016) B cell subsets (Figure 2) was observed in PHIV. This trend was verified in a smaller set of samples at T0 (Supplemental Figure 3B). No other differences were found in B cell subsets on samples collected at T0 (prevaccination), validating results shown at T2. Longitudinal variation of B cell subsets was also investigated. Both AM and TLM showed a significant contraction, in line with previous reports (22–24) (Supplemental Figure 3B). Additional analyses further revealed that AM B cells were significantly higher in late-treated PHIV (*P* = 0.049) compared with early-treated PHIV (Figure 2). To investigate MenB Ag-specific B cells, a probe targeting the fHbp Ag was used. A reduced frequency of fHbp^+^ SM B cells (CD27^+^IgD^–^IgG^+^fHbp^+^, *P* = 0.034) was found in children with PHIV, suggesting a potential perturbation of memory B cell generation (Figure 3A). A distinct distribution of fHbp^+^ B cells within maturational B cells subsets was observed in PHIV compared with HC. Indeed, a higher frequency of fHbp^+^ with AM or TLM characteristics (*P* = 0.038 and *P* = 0.051, respectively, Figure 3A), consistent with previous literature (25, 26), was found in PHIV. In this context, differences between early- and late-treated PHIV were also analyzed, and albeit not significant, a lower trend of fHbp^+^ SM frequency was observed in late-treated PHIV. Furthermore, the late-treated group was the only one demonstrating a decrease in fHbp^+^ SM compared with the HC group. This observation suggests that the late-treated group has the most substantial impact on the differences in cellular immunogenicity emerging between PHIV and HC (Figure 3A). Moreover, a similar trend was also found comparing SCs and NSCs, overall verifying the observed fHbp^+^ distribution in PHIV (Supplemental Figure 4). PHIV individuals exhibited a positive correlation between fHbp^+^ B cells in the SM subset and hSBA levels at T2, as well as a positive correlation between fHbp^+^ SM at T2 and the hSBA FC (Figure 3B). The association between prevaccination fHbp^+^ B cells and hSBA, because of higher likehood of meningococcal exposure in older children with PHIV, was also shown. Albeit not significant because of the small sample size, such an association was found only within fHbp^+^ SM B cells and not confirmed in AM or TLM fHbp^+^ cells. Distinct associations were observed between early- and late-treated PHIV (Figure 3C). The T2 fHbp^+^ SM B cells in late-treated PHIV were found to be positively correlated with the hSBA levels at T0. Conversely, the early-treated group showed a positive correlation between the fHbp^+^ SM B cells at T2 and both postvaccination hSBA levels and hSBA FC and a negative correlation between the fHbp^+^ TLM at T2 and the hSBA levels at T0 (Figure 3C). Additionally, the HC group showed a significant association between NadA IgG and hSBA FC (Supplemental Figure 5A), resembling the cross-antigen cooperative effect of the Ag vaccine and NadA’s pro-immune effects on Ag-presenting cells, which were reported in other studies (27, 28). This positive correlation was not present in both early- and late-treated PHIV (Supplemental Figure 5B).

### Gene expression of Ag-specific B cells may reveal distinct mechanisms of immunogenicity in PHIV.

**Figure 4:**
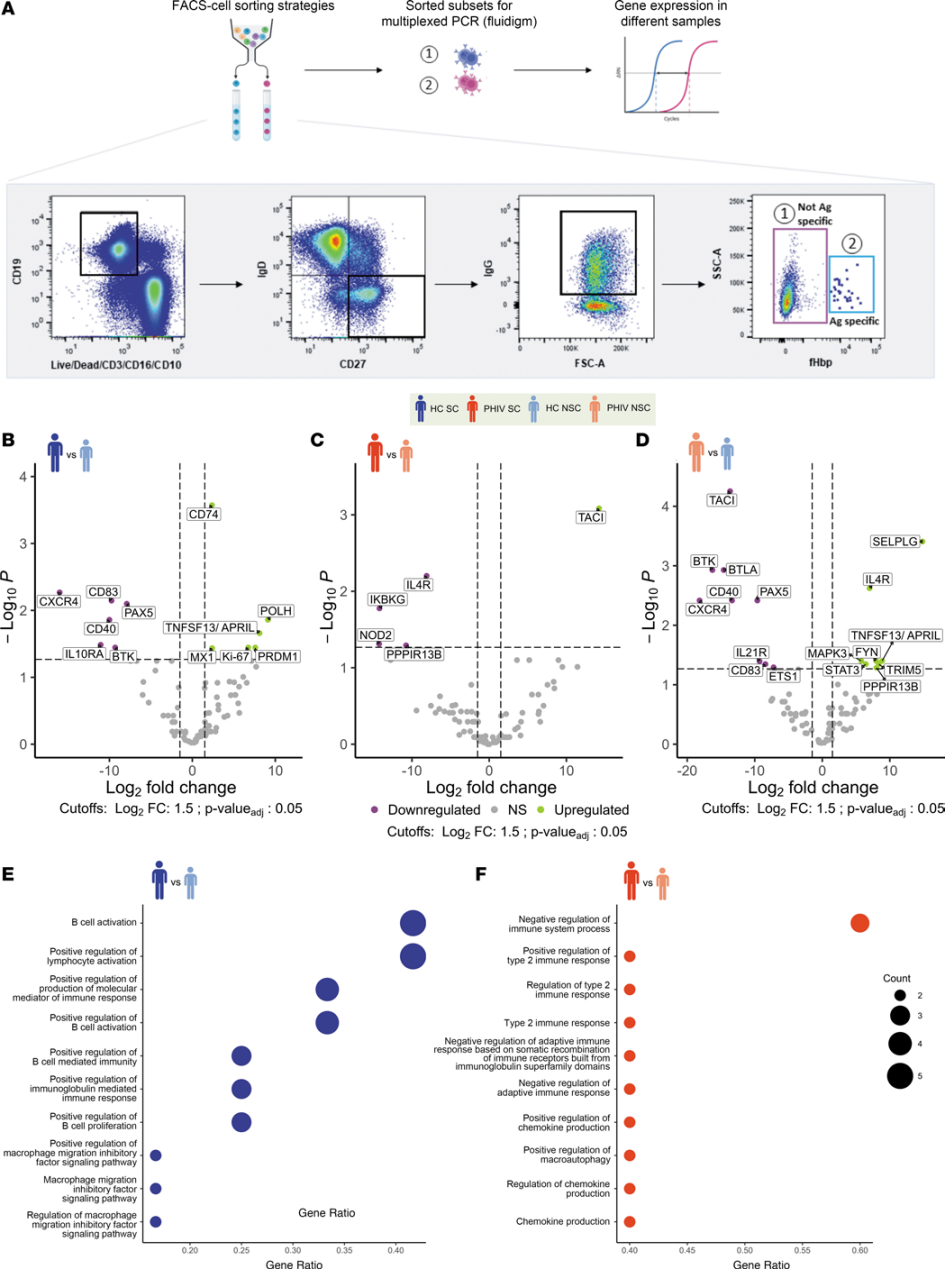
Mechanisms of vaccine responsiveness in PHIV and HC participants. The fHbp^+^ cells were sort-purified from PBMC samples in HC and PHIV participants at T2. Transcriptomic profiles of sort-purified fHbp^+^ cells from HC and PHIV PBMC samples were analyzed using the Fluidigm Biomark platform. (**A**) Experimental design. After sorting B cell subsets, gene expression is analyzed on Fluidigm Biomark. The cartoon on the top shows the experimental procedure. On the bottom are represented the gating strategies (black squares), and the B cell subsets selcted for sorting and gene expression analyses are indicated with numbers. (**B**–**D**) Volcano plots with the results of limma differential expression analyses in HC SC (*n* = 6) versus HC NSC (*n* = 4) (**B**), PHIV SC (*n* = 5) versus PHIV NSC (*n* = 3) (**C**), and PHIV NSC versus HC NSC (**D**), with upregulated genes in purple and downregulated in green. Benjamini-Hochberg–adjusted *P* values are reported. Vertical dashed lines mark FC cutoffs of ± 1.5. Horizontal dashed line marks FDR-adjusted *P* value cutoff of 0.05. (**E**) Dot plot reporting the Gene Ontology of DEGs in HC SC versus HC NSC comparison. (**F**) Dot plot reporting the Gene Ontology of DEGs in PHIV SC versus PHIV NSC comparison. Dot size represents the number of DEGs in each pathway.

To investigate the gene expression profile in relation to seroconversion after 2 doses of 4CMenB, B cell subsets along with non–Ag-specific B cells (CD27^+^IgD^–^IgG^+^fHbp^–^) and Ag-specific B cells (CD27^+^IgD^–^IgG^+^fHbp^+^) from samples collected at T2 were sorted, and their transcriptomic profile was analyzed by Fluidigm (Figure 4A). The results of the differential expression analyses are summarized in Supplemental Table 1. The fHbp^+^ SM B cell subset exhibited the highest number of differentially expressed genes (DEGs) when comparing PHIV and HC NSCs (18 DEGs) and no differences when comparing PHIV and HC SCs. Validating the distinct gene expression signature of NSCs in both groups, comparisons between SCs and NSCs in both HCs and PHIV revealed 12 and 5 DEGs, respectively. In HC SCs, both upregulated (*POLH*, *MX1*, *MKI67*, *PRDM1*, *APRIL*) and downregulated (*CD83*, *IL10RA*, *BTK*, *PAX5*, *CXCR4*) DEGs in fHbp^+^ SM cells were involved in the regulation of antibody production and memory response (Figure 4B). POLH polymerase is responsible for hypermutation during immunoglobulin class switch recombination (29), while PRDM1 is expressed in a specialized type of B cell responsible for antibody production (30). Additionally, the higher expansion of fHbp^+^ SM cells in the HC group could be attributed to the upregulation of proliferative genes, such as MKI67 (*Ki67*). These findings recapitulate the canonical molecular mechanisms underlying the antibody-mediated immune response. In contrast, in PHIV SCs, a downregulation of pro-inflammatory and apoptotic genes (*NOD2*, *IL4R*, *PPP1R13B*) was shown in association with an upregulation of *TACI*, well known to be involved in the antibody production and immune memory response (Figure 4C). Such a pro-inflammatory signature was further validated when comparing fHbp^+^ SM cells between the PHIV and HC groups of NSCs (Figure 4D). Furthermore, PHIV NSCs showed upregulation of additional pro-inflammatory genes (*SELPLG*, *TRIM5*, *STAT3*, *MAPK3*). Also, other B cell subsets were investigated at gene expression level and verified that comparisons within NSCs between HC and PHIV provided the largest number of DEGs (6, 12, and 12 DEGs, respectively, for naive B, total B, and SM B cells) (DEGs from all comparisons are available in Mendeley Data repository, doi: 10.17632/ztxfxh45p2.1). Overall, these findings suggest the presence of 2 distinct mechanisms of vaccine responsiveness in PHIV and HC, which was also supported by the pathways resulting from the Gene Ontology (GO) analyses of the DEGs in the 2 groups (Figure 4, E and F). Indeed, within PHIV SCs, DEGs were enriched (3/5 = 60%) in the negative regulation of the immune function pathway (GO:0002683) (Figure 4F). Conversely, within the HC SCs, DEGs were mainly enriched (5/12 = 42%) in B cell activation (GO:0042113) and positive lymphocyte regulation pathways (GO:0051251) (Figure 4E). In this context, to explore the impact of T cell immune activation and proliferation over time, T cell phenotype and HLA–DR^+^CD38^+^ CD4^+^ T cell levels were explored by flow cytometry in longitudinal samples (gating strategy in Supplemental Figure 6A). No major differences emerged in maturational T cell subsets between PHIV and HC. Both groups showed a significant increase of HLA–DR^+^CD38^+^ CD4^+^ T cells after 2 doses of 4CMenB vaccination (T2 vs. T0), which remained stable 1 year after vaccination (T3) (Supplemental Figure 6B). To investigate how 4CMenB immunogenicity is affected by the distribution of the T cell compartment, a correlation analysis across T cell subsets and hSBA was performed. Albeit limited by the small sample size, only HC demostrated a significant positive association between peripheral follicular helper T cells (pTfh cells, identified as CXCR5^+^ on central memory CD4^+^ T cells) at T2 and both hSBA at T2 and in terms of FC (T2–T0) (Supplemental Figure 7).

## Discussion

Vaccination has played a vital role in curbing the spread of infectious diseases. Ensuring successful 4CMenB vaccination of PLWH could be important for protection of these individuals. Children living with HIV have shown an increased susceptibility to bacterial (capsulated) infections in both high- and low-income countries (7, 8). As a result, immunization is a rational approach to reduce disease burden and unfavorable outcomes of infection. The strategic importance of 4CMenB immunization in the PHIV population has been further fueled by the recent evidence of its ability to confer cross-protection to *N*. *gonorrhoeae*, which causes an estimated 82 million cases/year (31, 32), especially in PLWH and men who have sex with men, in which the highest burden of sexually transmitted diseases is observed (13, 33). Despite both the European and the American Academy of Pediatrics guidelines recommending MenB vaccination in immunocompromised populations (34, 35), to date, only few studies have been conducted on these populations (14, 36, 37). In the present work, we report a deep immunophenotype characterization of the humoral, cellular, and transcriptomic 4CMenB vaccine-induced responses in PHIV, in a short- and long-term follow-up. As observed in other epidemiological studies (16, 17), we found a strong direct association between age and hSBA titers. At baseline, before 4CMenB vaccination, we observed a linear association between age of the participants and hSBA titers. This finding is in line with a recent observation in the UK population of an age-specific seroprevalence for meningococcus B reaching a peak between 30 and 39 years (16), suggesting multiple carriage episodes in teenagers and adults, with different strains over time. While HIV typically hinders an individual’s ability to generate optimal primary and secondary immune responses, as indicated in previous studies (38, 39), all HIV participants in this study achieved protective levels of MenB titers, comparable to the HCs. This aligns with recent findings in adults living with HIV (40, 41), where a good immune status at the time of vaccination and sustained virological suppression resulted in favorable immunogenicity following 4CMenB vaccination (13, 14). However, upon analyzing the magnitude of 4CMenB-induced humoral immune responses in this cohort of perinatally HIV-infected children, a notable reduction in the hSBA fold-increase was observed in the PHIV group compared with the HC group. Several vaccination studies in this population, and investigations into the memory B cell compartment, suggest that long-term serological memory may be impaired during HIV infection (25, 26). This is attributed to HIV exposure and viral replication following delayed ART treatment initiation. Accordingly, we examined the levels of hSBA and antibodies in participants with different timings of ART initiation (< 6 months vs. > 6 months). In line with previous findings with different vaccine Ags (20, 42), a higher magnitude of the hSBA response was observed in the early-treated group. The loss of vaccine-induced B cell memory begins in the early stages of HIV infection and becomes increasingly evident as the infection progresses to the chronic stage (26). In parallel a progressive expansion of atypical memory B cell subsets, including TLM and DN B cells, has been reported (43, 44).These heterogeneous B cell populations express an array of inhibitory receptors (FCRL4, CD22, and CD85j) and appear refractory to stimulation through their B cell receptor, TLRs, CD40, and cytokine receptors (24). This results in a diminished capacity for proliferation, affinity maturation, and the secretion of cytokines or antibodies, overall classifying such cells into the exhausted memory B cell compartment (45, 46). Moreover, it has been observed that HIV neutralization is significantly less effective in antibodies produced by atypical B cells compared with resting memory B cells (47) and that the hyporesponsive nature of these cells may contribute to the ineffective antibody responses generated by most PLWH against HIV and other pathogens (48, 49). In this study, we report, for the first time to our knowledge in the context of 4CMenB vaccination, an increased frequency of fHbp^+^ Ag-specific B cells in AM and TLM B cells, coupled with a reduction of Ag-specific SM B cells in PHIV. We further show that these dysregulated populations are linked to a reduced magnitude of humoral responses following 4CMenB vaccination. Accordingly, we have previously reported this phenomenon in the context of flu vaccination (50). In those studies, the analyses of the phenotype and gene expression from purified B cell subsets showed that perturbations in memory B cells persist in children living with HIV despite stable and long-term virological control, affecting the development and maintenance of vaccine-induced immune responses. HIV-infected individuals with apparently similar clinical and immunological characteristics can vary in responsiveness to vaccinations. However, molecular mechanisms responsible for such impairment, as well as biomarkers able to predict vaccine responsiveness in HIV-infected children, remain unknown. Our previous findings (50) suggest that in PLWH, the recovery achieved in overall B cell frequencies is not accompanied by recovery of gene expression and B cell function. Following the hypothesis that similar signatures may also inform immunogenicity response upon 4CMenB vaccination, we investigated this phenomenon at a transcriptomic level. We identified clear-cut differences in gene signatures of fHbp^+^ Ag-specific SM B cells between children living with HIV and their healthy peers unable to seroconvert after vaccination. The ability to downregulate genes involved in immune exhaustion/inflammation (*TACI*, *NOD2*, *IL4R*, *PPP1R13B*) discriminates between SCs and NSCs in PHIV, which allows HIV SCs to reach a transcriptional profile similar to HC SCs. Indeed, no DEGs were found between HIV and HC SCs within fHbp^+^ Ag-specific SM B cells. Conversely, a “classical” engagement of key genes required for the development of effective immune responses upon vaccination (*PRDM1*, *APRIL*, *CD74*, etc.) has been observed in the HC group. A distinct transcriptomic profile was reported in those participants who did not seroconvert upon 4CMenB vaccination. In HC, the response is predominantly achieved through canonical antibody production and memory response pathways whereas in PHIV, vaccine responsiveness is obtained through downregulation of the constitutive inflammatory state.

To further elucidate the contribution of the T cell compartment upon vaccination, longitudinal samples in both groups were investigated for maturational and activated T cell markers. An increase in HLA–DR^+^CD38^+^ CD4^+^ T cell frequency was observed in both groups following vaccination, albeit more pronounced in HC. These data support the reduced vaccine activation status observed in PHIV. In addition, a positive association between pTfh cells and 4CMenB hSBA titers was exclusively observed in HC, supporting the importance of such a T cell subset, as a peripheral surrogate of T-B cell interaction within the germinal center, to sustain immunogenicity. To our understanding, these insights contribute to the immunological alterations associated with HIV infection and highlight the importance of investigating the impact of HIV on B cell responses to vaccination, and investigations should be further focused on the Ag-specific level in the T cell counterpart. In conclusion, despite being effective and safe, the 2 doses of 4CMenB elicit distinct immune responses in PHIV in comparison with HC. Specifically, PHIV children showed a lower proportion of SM Ag-specific B cells and an increased frequency of Ag-specific atypical B cells coupled with a lower magnitude of specific humoral response upon vaccination. Larger data sets are needed to understand whether these alterations affect the long-term maintenance of protective immunity in this population.

## Methods

### Sex as a biological variable.

Our study examined male and female participants, and similar findings are reported for both sexes.

### Study design.

The study was conducted from September 2017 to November 2020 at Bambino Gesù Children’s Hospital in Rome and at “Luigi Sacco” Hospital in Milan. Eligible participants were in PHIV and HC groups and were aged 2–29 years. The inclusion criteria for PHIV were as follows: 2 independent PCR assays positive for HIV, HIV viral load < 40 copies/mL for at least 6 months, ART for at least 12 months, and no previous history of 4CMenB vaccination. Exclusion criteria for all participants were any history of meningococcal disease, chronic administration (defined as more than 14 days) of immunosuppressants or other immune-modifying medication within 3 months prior to the first vaccine dose or planned use thereof during the study, intravenous immunoglobulin administration within 90 days before the enrollment, and presence of lymphoproliferative or myelodysplastic diseases. Vaccination was deferred in the case of fever ≥ 38°C or acute illness on the day of intended vaccination or if the participant had received a dose of nonstudy vaccine within 2 weeks in the case of nonlive, or 4 weeks in the case of live, vaccines. Briefly, 2 doses of the 4CMenB vaccine were administered on day 1 (T0) and after 60 days (T1). A 0.5 mL dose of the multicomponent, recombinant serogroup B vaccine, 4CMenB (Bexsero, GSK), contained 50 μg of each of 3 purified *N*. *meningitidis* antigens (fHbp, NHBA, and NadA), 25 μg of outer membrane vesicle from *N*. *meningitidis* group B strain NZ 98/254 (containing PorA), and 1.5 mg aluminum hydroxide (EMA-Bexsero). The vaccine was administered by deep intramuscular injection in the deltoid arm muscle. EDTA blood samples (approximately 6 mL) were collected prior to the first dose administration (T0), 21 days after the second dose (T2), and 1 year later (T3). Plasma samples were obtained by centrifugation at 2,000*g* for 10 minutes and stored at –80°C until use. PBMCs were isolated by Ficoll density gradient centrifugation, resuspended in fetal bovine serum (FBS) supplemented with 10% DMSO, and cryopreserved in liquid nitrogen until use (51). A total of 63 PHIV and 28 HC participants were included in the analyses. A full report on samples assayed at every time point is reported in the repository file (Mendeley Data, doi: 10.17632/ztxfxh45p2.1). Additionally, data on the timing of ART initiation in the PHIV group were analyzed to compare the effect of early (within the first 6 months of life) versus late (at a later stage in life) treatment initiation on vaccine response.

### Safety.

Participants were monitored on site for 2 hours following each vaccination for any adverse event (AE). Solicited adverse events (SAEs) occurring during the 7 days following each vaccination were recorded in a diary card provided to participants and guardians. The monitored solicited local reactions were injection site pain, erythema, swelling, and induration, and the solicited systemic reactions were loss of appetite, nausea, myalgia, arthralgia, headache, and fever (body temperature ≥ 38°C). Moreover, unsolicited AEs were reported during the 7 days, and the intensity and relationship with the study vaccination of each recorded AE were determined by the study physician. The severity of the reaction was graded 1–4 according to the Division of AIDS (52). SAEs, medically attended AEs, reactions, and AEs that persisted beyond day 7 from vaccination, and AEs leading to study withdrawal, were collected throughout the study period.

### hSBA.

Humoral response to 4CMenB vaccination was measured through the standard hSBA. The hSBAs were performed at VisMederi Life Sciences Srl. Quantification was done as previously described (53). On day 1, an aliquot of the *N*. *meningitidis* serogroup B was plated and incubated overnight (ON) (16–18 hours) at 37°C with 5% CO_2_. The hSBA tests were performed on day 2 in 96-well, U-bottomed, microtiter plates. Previously heat-inactivated serum samples (56°C for 30 minutes) were added to the plates in a final concentration of 1:4, and 2-fold serial dilutions were performed. The bacterial colonies were harvested and resuspended in Dulbecco’s phosphate-buffered saline (DPBS) until they reached an optical density between 0.10 and 0.15 at 600 nm. The resulting cell suspension was then diluted in DPBS to reach about 50–60 CFU/10 μL. A total of 10 μL of the bacterial solution was added to each well together with 10 μL of a single donor human complement (Divbio Science), and different controls were performed in each plate (complement control, number of viable cells, serum samplesʼ complement-independent cell lysis). The plates were incubated for 1 hour at 37°C in a humidified CO_2_ incubator. After that, a 10 μL drop from each well was taken and plated on an agar plate, which was subsequently incubated ON at 37°C with 50% CO_2_. On day 3, the colonies of each condition were counted and verified. The serum bactericidal titer was reported as the reciprocal serum dilution yielding at least 50% killing as compared with the number of viable cells measured in the control. To assign a titer, the test sera must have a clear hSBA titer, and to determine a 50% CFU reduction titer, at least 2 consecutive serial dilutions of the test serum must have cell counts < 20% CFU compared with the number of viable cells in the control. According to WHO indications, vaccine responders were defined as participants who seroconverted (≥4-fold increase on the baseline hSBA titer) and the nonresponders as NSCs (hSBA increase < 4-fold), whereas study participants with a titer ≥ 4 were considered seroprotected (54). hSBA FC was calculated by dividing hSBA at T2 by hSBA at T0.

### Serology.

Quantification by Oxford Vaccine group of IgG titers to Ags of capsular group B *N*. *meningitidis* (fHbp, NadA, and NHBA) was performed using a standard “sandwich” ELISA. Microtiter plates coated with fHbp, NadA, and NHBA Ags were incubated with sera from study participants to allow binding of specific antibodies to the Ag. Next, a secondary polyclonal anti-human IgG antibody (Jackson ImmunoResearch 109-055-003) was added. Between each step, the plates were washed with a mild detergent solution to remove nonspecifically bound antibodies. Following the final wash step, the plate was developed by adding an enzymatic substrate that reacted with the phosphate, resulting in a colored product. The intensity of the color correlated with the amount of antibody present in the sera sample. Serum antibody concentrations were then calculated by means of the standard curve.

### Group B meningococcus–specific memory B cell analyses.

Cryopreserved PBMCs were thawed and resuspended in R10 medium (RPMI 1640 and 10% FBS supplemented with 1% penicillin/streptomycin and l-glutamine). After washing, they were resuspended in FACS Wash Buffer (2% FBS in DPBS) and stained with Live/Dead (BV510) to assess viability and with surface antibodies CD3-BV510 (BD Biosciences 563109), CD16-BV510 (BD Biosciences 563820), CD10-BV510 (BD Biosciences 659121), CD19-APC-R700 (BD Biosciences 563032), IgD-BV421 (BD Biosciences 565940), CD27-FITC (BD Biosciences 555440), IgG-BV610 (BD Biosciences 563246), CD21-APC (BD Biosciences 559867), and CD38-PE-Cy7 (BD Biosciences 335825) to define maturational B cell subsets. Moreover, Lightning-Link R-PE Conjugation Kit (Innova Biosciences) was used to obtain fHbp^+^ PE probe as previously described (39) to identify Ag-specific B cells. Aliquots of 100 total B cells (live, singlets, CD19^+^), naive B cells (IgD^+^CD27^–^), Ag-specific B cells (IgD^–^CD27^+^IgG^+^fHbp^+^), and non–Ag-specific IgD^–^CD27^+^IgG^+^fHbp^+^ B cells were sort-purified by FACSAria II (BD Biosciences). Distribution of fHbp^+^ B cells was carried out by gating into TLM (CD21^–^CD27^–^), resting memory (CD21^+^CD27^+^), and AM (CD21^–^CD27^+^) subsets. The purity of the sorted cell population was 99.9%. One hundred live cells per B cell subset were sorted in PCR tubes previously loaded with 9 μL of CellsDirect 1-step PCR buffer and pooled TaqMan gene expression assays (2× CellsDirect Reaction mix 5 μL, Superscript III + Taq polymerase 0.5 μL, 0.2× TaqMan primer pool 2.5 μL, Resuspension Buffer 1 μL; Invitrogen, Thermo Fisher Scientific). After sorting, samples were reverse-transcribed, and target-specific preamplification was performed on a C1000 Thermal Cycler (Bio-Rad) with the following scheme: 50°C for 20 minutes, 95°C for 2 minutes, 95°C for 15 seconds, and 60°C for 4 minutes, with the last 2 steps repeated for 18 cycles. Resulting cDNA was stored at −20°C until further analysis.

### T cell analyses.

Cryopreserved PBMCs were thawed and resuspended in R10 medium. After washing, they were resuspended in R10 medium to a final concentration of 4 × 10^6^ cells/mL, plated in a 48-well plate, and left ON at 37°C to recover. The following day, cells were collected, washed, and resuspended in FACS Wash Buffer (2% FBS in DPBS), then stained with Live/Dead (UV440) 15 minutes at room temperature to assess viability. Then, the cells were stained with CXCR5-APC for 20 minutes at 37°C and afterward stained for 30 minutes at 4°C with the surface antibodies CD38-FITC (BD Biosciences 555459), CXCR5-APC (BD Biosciences 558113), CD45RA-APC-H7 (BD Biosciences 560674), HLA-DR-BUV395 (BD Biosciences 565972), CD3-BUV496 (BD Biosciences 612941), CD27-BUV563 (BD Biosciences 748705), CD127-APC-R700 (BD Biosciences 565185), CD4-BV786 (BD Biosciences 563881), CD25-PE-Cy5.5 (Invitrogen, Thermo Fisher Scientific, 35-0259-42), and PD1-BV421 (BD Biosciences 562516) to define T cell subsets and frequencies. The samples were acquired on BD Biosciences FACSymphony A3.

### Multiplexed real-time PCR.

Previously amplified samples (cDNA) were loaded on a Fluidigm 96.96 standard chip following manufacturer’s instructions. Briefly, an assay premix was prepared at 1:1 — 20× TaqMan Gene Expression Assay (Applied Biosystems) and Assay Loading Reagent (Fluidigm, Biomark). The sample premix was prepared with TaqMan Universal PCR Master Mix (2×) (Applied Biosystems), 20XGE Sample Loading Reagent (Fluidigm), and preamplified cDNA. Finally, 5 μL of assay and sample mix was loaded into the chip. The list of the probes in the panel is the same as previously described. Raw files containing cycle threshold (Ct) values were quality-checked, and low-quality samples were filtered out of the analyses. Data from multiple arrays were cyclic loess normalized with limma (55). Expression threshold values were finally obtained as 40-Ct to be further used for the final analyses, as previously described (56). The resulting gene expression profile was analyzed with limma, selecting DEGs based on adjusted *P* ≤ 0.05 and FC ≥ 1.5.

### Statistics.

Statistical analyses were performed using R (version 4.2.2) and GraphPad Prism (version 9). Missing data were excluded from the analyses, and the selection of the most appropriate statistical test was performed according to data distribution. Normality assumption was tested by the Shapiro-Wilk test, and homogeneity of variance was validated by Levene’s test. Differences between groups were assessed using the Welch *t* test (2 tailed) or ANOVA (1 way when comparing between > 2 groups or 2 way when comparing between > 2 groups and time points) when normality and homoscedasticity assumptions were met. Alternatively, the nonparametric Mann-Whitney *U* test or Kruskal-Wallis test was employed when normality assumption was not satisfied. Post hoc analyses were conducted using pairwise *t* test (2 tailed) and Dunn’s test as appropriate, and paired tests were used for longitudinal analyses. Differences between categorical data were evaluated via Fisher’s χ^2^ test. Correlations between variables were assessed using Pearson’s correlation coefficient (*r*). *P* values ≤ 0.05 were considered significant, and the Benjamini-Hochberg correction was applied to control the FDR when multiple comparisons were performed. GO analysis was performed using clusterProfiler (version 4.6.0). Effect size for sample size power calculations was performed based on the observed data of seroconversion after vaccination. To achieve an 80% power at a significance level of 0.05, 31 patients per group would be needed.

### Study approval.

The study was conducted in compliance with the Declaration of Helsinki and received approval from the ethics committees of Bambino Gesù Children’s Hospital, Rome, Italy (approval number: 1497_OPBG_2017), and “Luigi Sacco” Hospital, Milan, Italy (approval number: 7149_2019). Informed written consent was obtained prior to participation from all the study participants or their parents/legal representatives.

### Data availability.

Original data and data supporting the findings of this study are available on Mendeley Data (doi: 10.17632/ztxfxh45p2.1). Values for all data points shown in graphs are reported in the Supporting Data Values file. Scripts to reproduce the analyses presented in each figure of the paper are available at https://github.com/palma-lab/4CMenB-manuscript (commit ID 5425949).

## Author contributions

NC, AN, MS, VS, ECM, AR, and PP verified the data underlying the manuscript. NC, PP, AJP, and E Montomoli conceived the study; NC, ECM, VS, and AR developed methodology; MS and AN developed software and visualization; MS, AN, and NC performed formal analyses; NC, AN, MS, VS, ECM, GRP, E Morrocchi, DA, AR, MLCDA, IB, SG, IC, VC, DT, GO, SB, DO, E Montomoli, AJP, and PP investigated; AJP, PP, and DC provided supervision and resources; MS, AN, and GO prepared the original draft; NC, AN, MS, VS, ECM, GRP, E Morrocchi, DA, AR, MLCDA, IB, SG, IC, VC, DT, GO, SB, DO, E Montomoli, AJP, and PP reviewed and edited; and PP acquired funding. Co–first authors were ordered alphabetically. All authors have read and agreed to the published version of the manuscript.

## Supplementary Material

Supplemental data

Supporting data values

## Figures and Tables

**Table 1:**
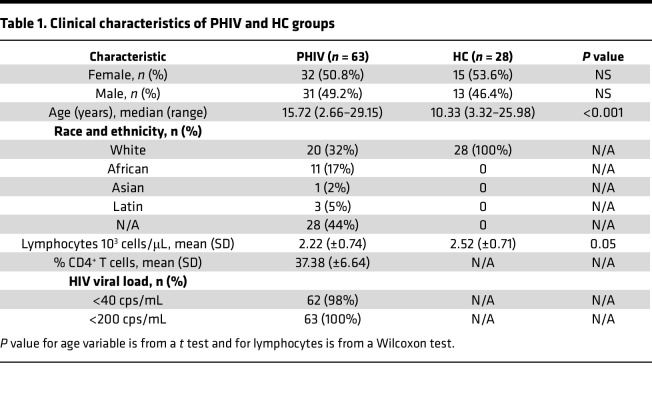
Clinical characteristics of PHIV and HC groups

## Version 1 05/22/2024

Electronic publication
