# Supplementary material for: Children with perinatally acquired HIV exhibit distinct immune responses to 4CMenB vaccine
Source: JCI Insight. 2024 May 22;9(10):e177182. doi: 10.1172/jci.insight.177182 (PMC11141905; doi:10.1172/jci.insight.177182)
Supplement: Supplemental data [file jciinsight-9-177182-s089.pdf]

SUPPLEMENTARY DATA

Supplementary Table 1. Limma DEGs results

Cell type	Cell subset	Conditions				
		HIV vs HC	HIV (SC vs NSC)	HC (SC vs NSC)	HIV vs HC (SC)	HIV vs HC (NSC)
B	Naïve	6	4	0	5	6
	CD19	5	1	6	3	12
	SM	1	0	9	2	12
	fHbp+ SM	1	5	12	0	18

Table reporting the number of DEGs resulting from limma analysis. Foxp1 gene was excluded from the analysis due to a low fitness of the probe. Full list of DEGs is reported in the Mendeley repository (link).

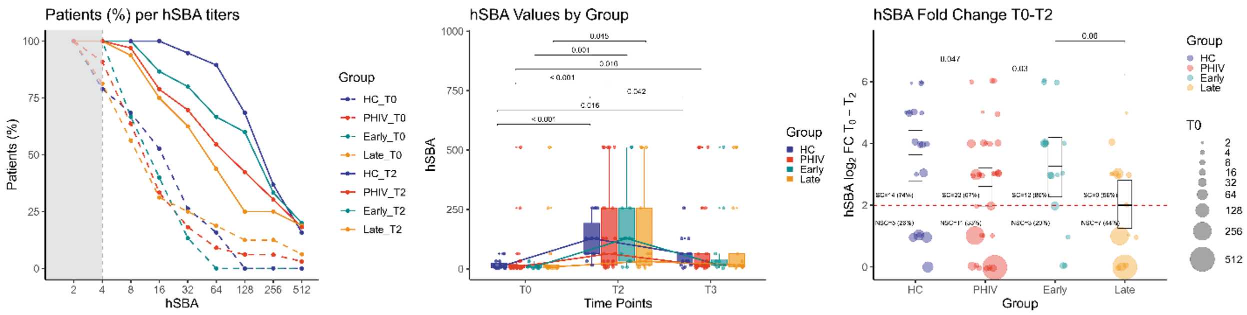

Supplementary Figure 1. 4CMenB vaccine elicited hSBA response in age-matched PHIV and HC participants. hSBA titers were measured in the sera of participants at T0, T2 and T3. Age matched analyses are reported. **(A)** Line plot reporting the percentage of participants per hSBA levels at T0 (dashed lines) and T2 (solid lines) for HC (n = 19, blue) and PHIV (n = 33, red) groups, early- (n = 15, cyan) and late- (n = 16, orange) treated PHIV individuals. Gray dashed line marks the threshold of Seroprotection (SP: hSBA T0 = 4) with gray area showing NSP (Non-seroprotected). **(B)** Longitudinal analysis of hSBA titer (± 95% C.I.) in HC (n = 15, blue), PHIV (n = 25, red), Early- (n = 12, cyan) and Late- (n = 13, orange) treated individuals pre-(T0) and post-vaccination (T2-T3). The lines represent the median values of HC (blue), PHIV (red), Early- (cyan) and Late- (orange) treated individuals. **(C)** Log2 hSBA titer Fold Change (FC) (± 95% C.I.) in HC (n = 19, blue), PHIV (n = 33, red), in Early- (n = 15, cyan) and Late- (n = 16, orange) treated participants following vaccination. The size of the points represents the hSBA value at T0. The dashed line represents the threshold of protection (FC ≥ 4); *t*-test if both distributions were normal, or conversely, with Kruskal-Wallis test followed by Dunn's test. FDR adjusted p-values < 0.05 are reported.

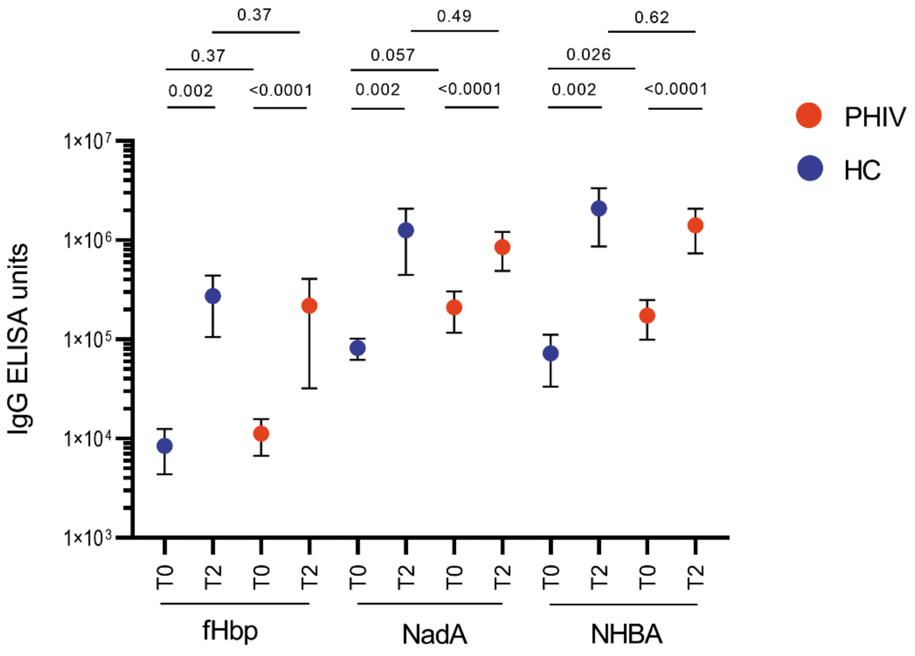

Supplementary Figure 2. 4CMenB vaccine elicited serological responses in PHIV and HC participants.

IgG titers specific to fHbp, NadA, and NHBA antigens were measured in the sera of HC (n = 13) and PHIV (n = 20) participants at T0 and T2. hSBA titers measured in the sera of participants at T0 were correlated with the age of each subject. Mean IgG levels (± 95% C.I.) for three antigens (fHbp, NadA and NHBA) measured by ELISA are reported. All comparisons were tested by two-tailed Mann-Whitney *U* test.

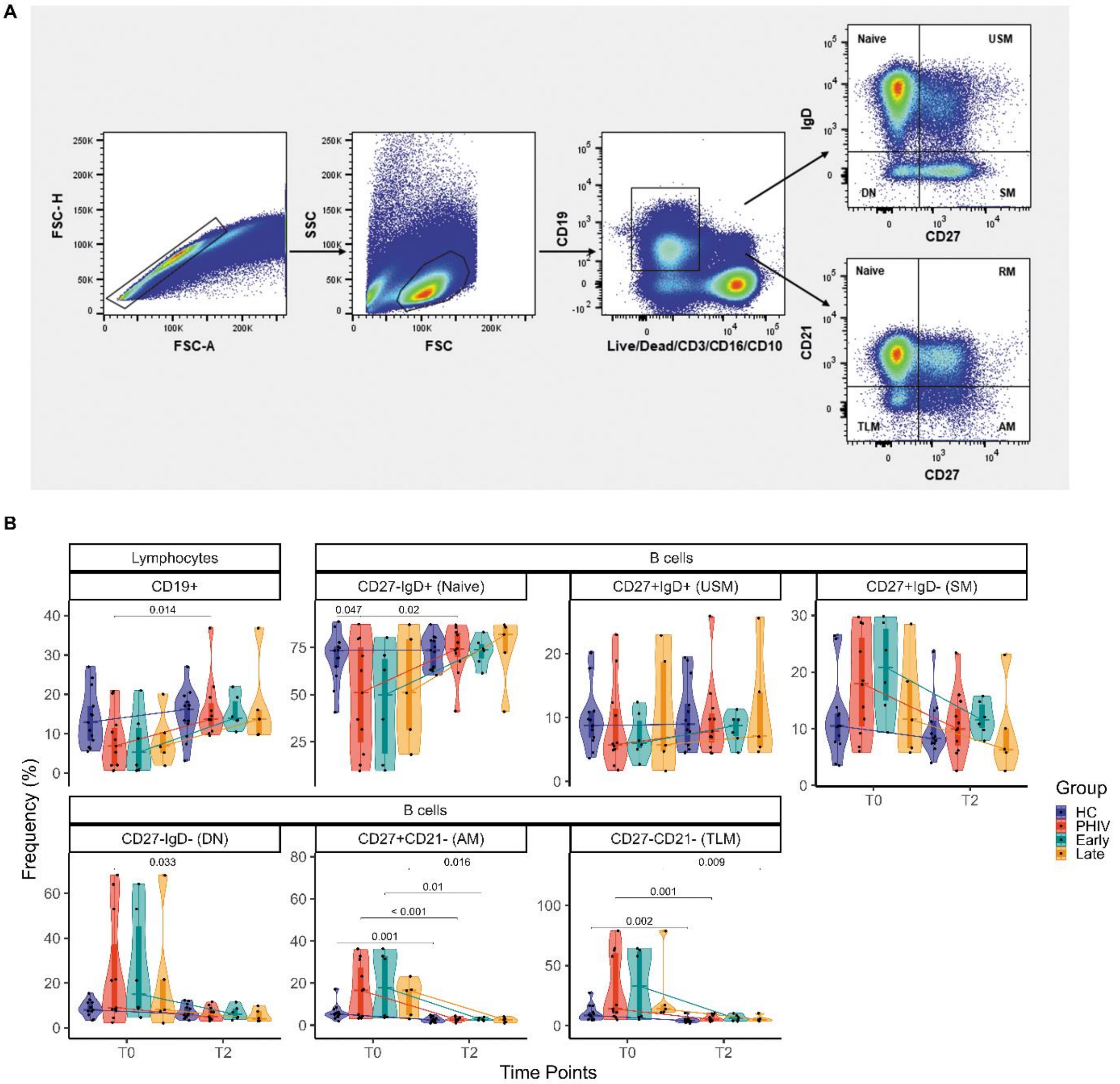

Supplementary Figure 3. Gating strategy of B cells subsets and longitudinal analysis of B cell subsets in PHIV and HC participants.

(A) Gating strategy for B cells subsets. **(B)** Violin and box plots of the longitudinal frequencies of B cell subsets at T0 and T2 measured in HC (n = 13), PHIV (n = 11), Early (n = 6), and Late (n = 5) -treated participants. Boxplot midlines report the median, with the upper and lower limits of the box being 75th and 25th percentile respectively. Whiskers represent 1.5 IQR. Comparison of hSBA distributions between groups and time points was evaluated by ANOVA followed by pairwise *t*-test if both distributions were normal, or conversely, with Kruskal-Wallis test followed by Dunn's test. FDR adjusted p-values < 0.05 are reported.

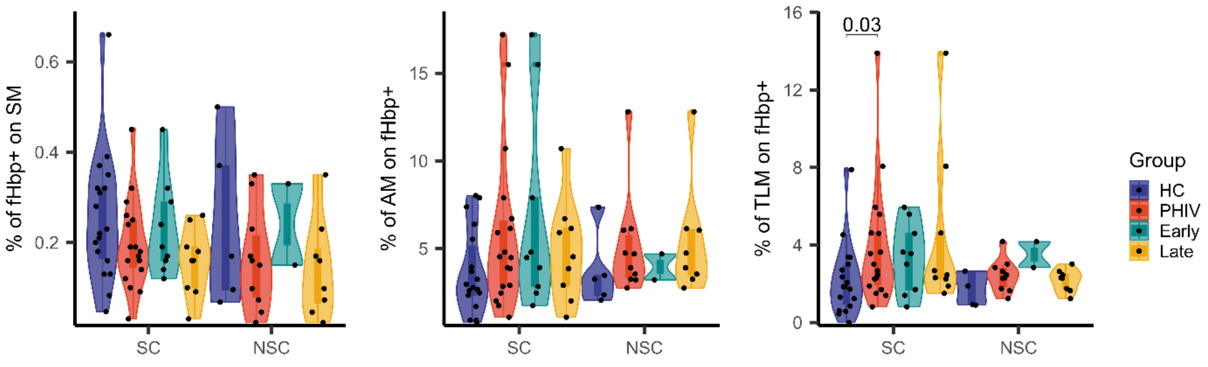

Supplementary Figure 4. Ag-specific B cell subsets distribution in SC and NSC

Violin and box plots of the frequencies of Ag-specific (fHbp+) B cells measured at T2 in SC and NSC of PHIV (n_{SC} = 18, n_{NSC} = 10, red), HC (n_{SC} = 18, n_{NSC} = 5, blue), Early- (n_{SC} = 9, n_{NSC} = 2, cyan) and Late- (n_{SC} = 9, n_{NSC} = 8, orange) treated PHIV participants. Boxplot midlines report the median, with the upper and lower limits of the box being 75th and 25th percentile respectively. Whiskers represent 1.5 IQR. Comparison of fHbp+ distributions in SC and NSC between groups was evaluated by ANOVA followed by pairwise t-test if both distributions were normal, or conversely, with Kruskal-Wallis test followed by Dunn’s test. FDR adjusted p-values < 0.05 are reported. Comparison of fHbp+ distributions within Groups was evaluated by t-test if both distributions were normal, or conversely, with Wilcox test.

A

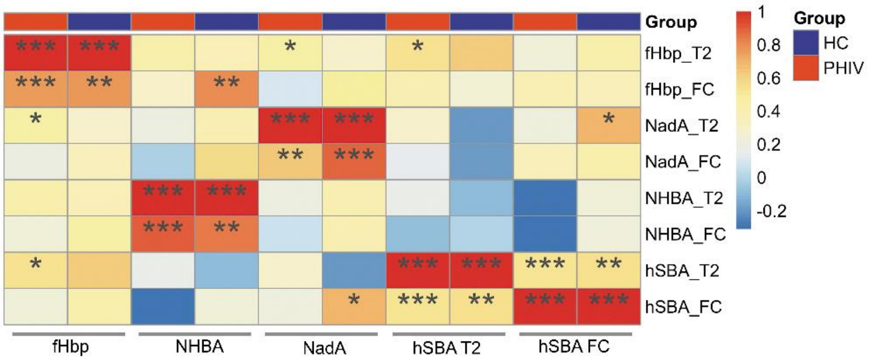

B

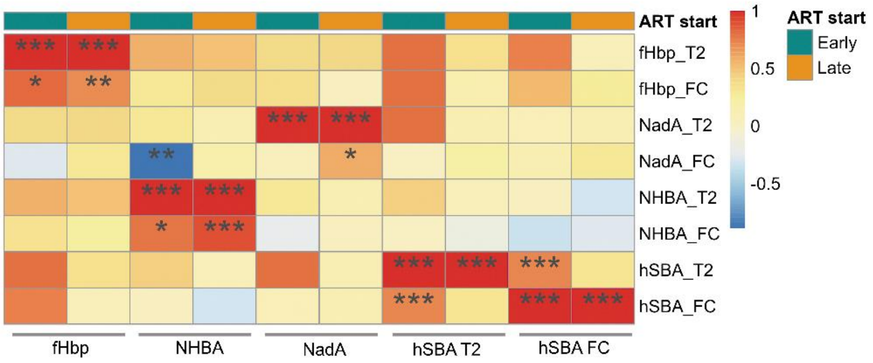

Supplementary Figure 5. Immunological correlates in PHIV and HC participants.

(A) Pearson Correlation heatmap between IgG levels against the different 4CMenB antigens (fHbp, NadA, NHBA) and hSBA values in HC (n = 26) and PHIV (n = 59) at T2. **(B)** Pearson Correlation heatmap between IgG levels against the different 4CMenB antigens (fHbp, NadA, NHBA) and hSBA values in Early- (n = 21) and Late- (n = 31) treated PHIV at T2. P-value ≤ 0.05 (*), ≤ 0.01 (**), ≤ 0.001 (***).

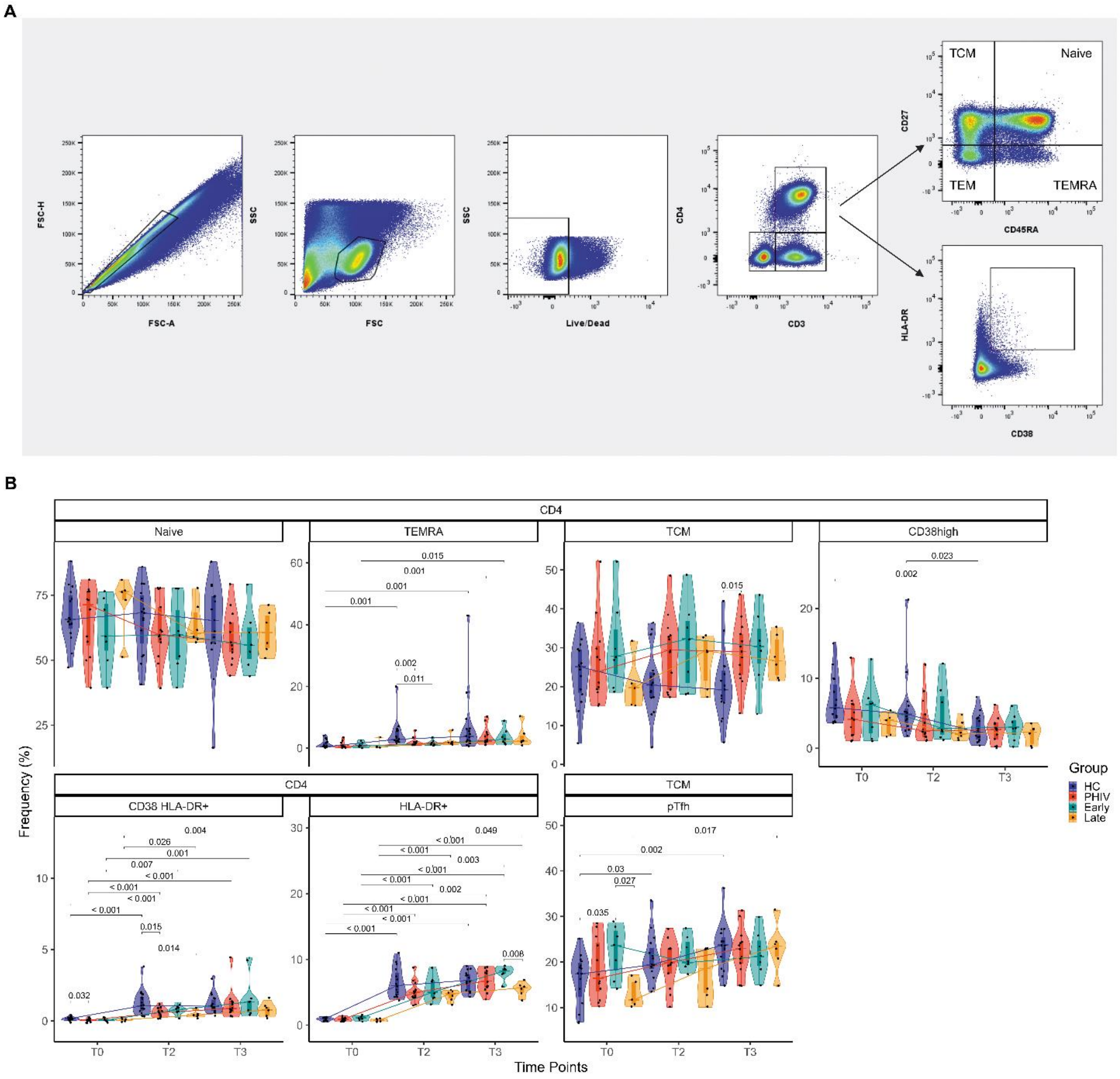

Supplementary Figure 6. Gating strategy of B cells subsets and longitudinal analysis of T cell subsets in PHIV and HC participants.

(A) Gating strategy for T cells subsets. **(B)** Violin and box plots of the longitudinal frequencies of T cell subsets (Naïve; TCM, T Central Memory; TEMRA, T Effector Memory Expressing CD45RA) at T0, T2 and T3 measured in HC ($n_{T0, T2, T3}$ = 15), PHIV (n_{T0} = 12, $n_{T2, T3}$ = 13), Early ($n_{T0, T2, T3}$ = 7), and Late (n_{T0} = 5, $n_{T2,T3}$ = 6) -treated participants. pTfh cells were gated as CXCR5+ cells on TCM. Boxplot midlines report the median, with the upper and lower limits of the box being 75th and 25th percentile respectively. Whiskers represent 1.5 IQR. Comparison of hSBA distributions between groups and time points was evaluated by ANOVA followed by pairwise *t*-test if both distributions were normal, or conversely, with Kruskal-Wallis test followed by Dunn’s test. FDR adjusted p-values < 0.05 are reported.

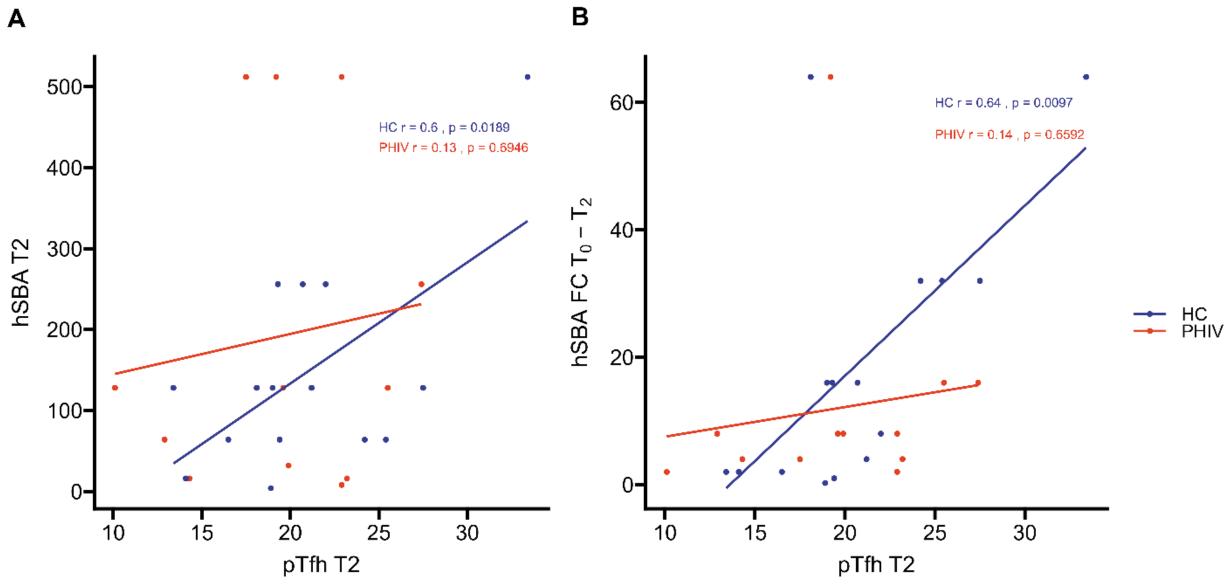

Supplementary Figure 7. Correlation plots between pTfh cells and hSBA

Pearson correlation analyses between hSBA values and frequencies of pTfh on TCM T cells at T2. Regression line is reported in blue for HC (n = 15) and red for PHIV (n = 12). (A) Pearson correlation between hSBA at T2 and % of pTfh on TCM T cells. (B) Pearson correlation between hSBA FC and % of pTfh on TCM T cells.
